# Surface Functionalization of Nanoparticles for Enhanced Electrostatic Adsorption of Biomolecules

**DOI:** 10.3390/molecules30153206

**Published:** 2025-07-30

**Authors:** Marks Gorohovs, Yuri Dekhtyar

**Affiliations:** Mechanical and Biomedical Engineering Institute, Riga Technical University, Kipsalas Street 6B, LV-1048 Riga, Latvia; jurijs.dehtjars@rtu.lv

**Keywords:** nanoparticles, biomolecules, drugs, drug–nanoparticle conjugates, adsorption processes, proteins, lipids, electrostatic interaction, functionalization

## Abstract

Electrostatic adsorption plays a crucial role in nanoparticle-based drug delivery, enabling the targeted and reversible loading of biomolecules onto nanoparticles. This review explores the fundamental mechanisms governing nanoparticle–biomolecule interactions, with a focus on electrostatics, van der Waals forces, hydrogen bonding, and protein corona formation. Various functionalization strategies—including covalent modification, polymer coatings, and layer-by-layer assembly—have been employed to enhance electrostatic binding; however, each presents trade-offs in terms of stability, complexity, and specificity. Emerging irradiation-based techniques offer potential for direct modulation of surface charge without the addition of chemical groups, yet they remain underexplored. Accurate characterization of biomolecule adsorption is equally critical; however, the limitations of individual techniques also pose challenges to this endeavor. Spectroscopic, microscopic, and electrokinetic methods each contribute unique insights but require integration for a comprehensive understanding. Overall, a multimodal approach to both functionalization and characterization is essential for advancing nanoparticle systems toward clinical drug delivery applications.

## 1. Introduction

Effective drug delivery is one of the most critical challenges in modern medicine. Despite significant advances in pharmaceutical development, over 90% of new drug candidates fail to reach the market, often due to poor bioavailability, instability in biological fluids, or inadequate targeting of diseased tissues [1]. Traditional drug administration methods, such as oral or intravenous delivery, frequently suffer from non-specific distribution, leading to off-target effects, toxicity, and reduced therapeutic efficacy. As a result, developing more efficient and targeted drug delivery systems is crucial for enhancing treatment outcomes, minimizing side effects, and facilitating the use of fragile or potent therapeutic agents.

Nanoparticles (NPs) have emerged as highly promising carriers in this field due to their small size, large surface-to-volume ratio, and controllable surface properties. These features allow nanoparticles to circulate in the bloodstream, penetrate biological barriers, and deliver drugs in a controlled and targeted manner [2,3]. One of the key aspects of nanoparticle-based drug delivery systems is their ability to adsorb biomolecules, including therapeutic proteins, nucleic acids, and targeting ligands, to enhance their interaction with specific cells or tissues.

Biomolecules can be attached to NPs surfaces through covalent or non-covalent interactions. Covalent bonding provides stable and long-lasting attachment of biomolecules, while non-covalent interactions enable reversible and stimuli-responsive loading [4,5]. Among the non-covalent interactions, electrostatic adsorption has gained significant attention due to its simplicity, tunability, and reversible nature. Despite advancements in NP-based drug delivery, achieving controlled and high-affinity biomolecule loading remains challenging, as it often leads to unwanted aggregation, rapid clearance, or off-target effects [6]. Enhancing the electrostatic interactions between nanoparticles and biomolecules through surface functionalization presents a promising strategy to overcome these challenges.

This review aims to provide an overview of the mechanisms of electrostatic adsorption in drug delivery, discuss recent advances in surface functionalization strategies to enhance these interactions, and examine the characterization techniques used to study nanoparticle–biomolecule interactions. In particular, it emphasizes emerging methods such as irradiation-based surface modification, which offer reagent-free alternatives for surface charge modification. A distinctive feature of this review is the integration of both functionalization approaches and characterization techniques, which are often treated separately but are interdependent in practice. By presenting functionalization and characterization strategies within a unified framework, this review seeks to bridge existing knowledge gaps and support the rational design of nanoparticle systems with improved electrostatic adsorption of biomolecules.

## 2. Fundamentals of Nanoparticle–Biomolecule Interactions

The adsorption of biomolecules onto nanoparticles (NPs) is governed by a complex interplay of physical and chemical forces operating at the nano–bio interface. These include electrostatic interactions, van der Waals forces, hydrogen bonding, hydrophobic effects, and steric hindrance. Understanding these mechanisms is crucial for designing effective surface functionalization strategies that promote the selective and stable binding of biomolecules, such as proteins, DNA, or peptides.

### 2.1. Electrostatic Interactions

Electrostatic forces are often the dominant contributor to nanoparticle–biomolecule adsorption, particularly in aqueous environments. These forces arise from the attraction between oppositely charged surfaces, as described by Coulomb’s law. Their strength and direction are susceptible to the properties of the surrounding medium, which influence the ionization state of surface groups and the thickness of the electric double layer. Key environmental factors that modulate electrostatic interactions include pH, ionic strength, and temperature [7]. The isoelectric point (pI) of a biomolecule is the pH at which it carries no net charge. At pH values above the pI, biomolecules acquire a negative charge, while below the pI, they become positively charged. This charge behavior determines whether attraction or repulsion occurs with a given nanoparticle surface [8,9]. Increasing ionic strength leads to charge screening and compression of the electric double layer, which can diminish long-range electrostatic interactions and hinder adsorption [9,10]. Temperature can further influence electrostatics by altering the dielectric constant of water, the conformational flexibility of biomolecules, and diffusion kinetics, potentially enhancing or disrupting adsorption depending on system stability [11]. Electrostatic forces are relatively long-range and tunable, making them highly useful for engineering nanoparticle surfaces to promote selective and stable adsorption of biomolecules.

### 2.2. Van der Waals and Hydrophobic Interactions

Van der Waals forces, though relatively weak and non-specific, become important when particles and molecules are in close proximity. These interactions result from transient dipole attractions and contribute to the baseline affinity in nearly all nano–bio systems [12,13]. Adsorption is also influenced by entropy: displacement of structured water or counterions can create an entropic gain that favors binding. At the same time, conformational restrictions on the biomolecule may reduce the overall free energy benefit. For instance, hydrophobic interactions occur when nonpolar molecules or particles cluster together in aqueous environments to minimize their exposure to water. It plays a significant role, particularly with nonpolar nanoparticle surfaces such as graphene oxide or hydrophobic polymers [14,15].

### 2.3. Hydrogen Bonding and Specific Interactions

Hydrogen bonding adds a layer of specificity and directionality to NP–biomolecule binding. Functional groups, such as hydroxyls, carboxyls, and amines, on the nanoparticle surface can form hydrogen bonds with complementary sites on biomolecules, thereby stabilizing the adsorption [16,17]. In certain systems, specific interactions such as ligand–receptor binding or π–π stacking also contribute to affinity and selectivity [18,19]. However, if the surface is tightly packed with molecules, polymer chains, or functional groups, steric hindrance may occur. It can prevent biomolecules from accessing the nanoparticle surface. While steric stabilization is beneficial for colloidal stability, it may limit electrostatic interactions by physically blocking binding sites [20,21].

### 2.4. DLVO Theory and Colloidal Stability

The Derjaguin–Landau–Verwey–Overbeek (DLVO) theory provides a framework for understanding colloidal interactions by balancing van der Waals attraction with electrostatic repulsion. In nanoparticle systems, this theory helps predict aggregation behavior and conditions under which biomolecules are likely to adsorb or be repelled. For instance, increasing ionic strength and temperature compresses the electrical double layer, reducing electrostatic repulsion and promoting adsorption or aggregation [22,23].

### 2.5. Protein Corona Formation

Upon exposure to biological fluids, nanoparticles rapidly adsorb a dynamic layer of biomolecules, predominantly proteins, forming what is known as the protein corona. This corona defines the nanoparticle’s biological identity and affects its fate in vivo, influencing cellular uptake, biodistribution, and immune response. The protein corona consists of two layers: a hard corona of tightly bound proteins and a soft corona of loosely associated ones. The composition of the corona is highly dependent on surface charge, hydrophobicity, and environmental conditions [24,25]. Electrostatic interactions play a central role in its initial formation, especially when the NP surface is functionalized to favor specific charge-mediated binding.

## 3. Surface Functionalization Approaches to Enhance Electrostatic Adsorption

Surface functionalization is crucial for controlling the electrostatic properties of nanoparticles, thereby enabling improved and selective adsorption of charged biomolecules. Functionalization strategies not only influence the magnitude of surface charge but also affect colloidal stability, biocompatibility, and loading capacity. Below are the major strategies used to impart or tune surface charge and functionality for this purpose.

### 3.1. Direct Chemical Functionalization

Direct chemical functionalization refers to the covalent modification of nanoparticle surfaces using small charged molecules or reactive groups. This approach enables precise control over the type, density, and orientation of functional groups, such as carboxyl (–COOH) [26], amine (–NH_2_) [27,28], or thiol (–SH) [29], which directly influence electrostatic interactions with biomolecules. A commonly used method for silica and metal oxide nanoparticles is silanization, where organosilanes, such as (3-aminopropyl)triethoxysilane (APTES), are grafted to the surface, introducing positive charges [30,31]. Conversely, carboxylic acid-functionalized silanes (e.g., carboxyethylsilanetriol) provide negatively charged surfaces suitable for adsorbing positively charged proteins or peptides [26].

Carbon-based nanomaterials such as graphene oxide (GO) or carbon nanotubes (CNTs) can be oxidized with strong acids to introduce oxygen-containing groups, rendering them negatively charged and highly reactive [32,33]. Functionalization with sulfonic acids [34,35] or phosphate groups [34,36] has also been reported to enhance bioactivity and colloidal stability. Additionally, click chemistry and bioorthogonal reactions, such as azide–alkyne cycloaddition, enable the efficient and site-specific attachment of charged ligands or peptides onto NP surfaces [37]. These approaches are increasingly utilized in bioconjugation and targeted drug delivery systems, where controlled electrostatic interactions are crucial, as they provide robust control but may be limited by factors such as irreversible binding and chemical instability under physiological conditions.

### 3.2. Polymer Wrapping and Coating

Polymer wrapping significantly alters the surface electrostatic potential of nanoparticles, making it a widely used strategy for enhancing selective adsorption of oppositely charged biomolecules. It involves coating the nanoparticle surface with charged or amphiphilic polymers that modify its surface potential and provide multivalent interaction sites. Cationic polymers, such as polyethyleneimine (PEI) [38,39], chitosan [40], and poly(L-lysine) [41], are commonly employed to render the NP surface positively charged, thereby enhancing the adsorption of negatively charged biomolecules, including DNA, RNA, and acidic proteins. These coatings also stabilize the nanoparticles in aqueous media and prevent aggregation through steric repulsion [42].

Anionic polymers, such as poly(acrylic acid) (PAA) [43] and poly(styrene sulfonate) (PSS) [44], are used to create negatively charged surfaces, which promote binding to cationic antibiotics. The conformation, density, and molecular weight of polymers influence the accessibility of functional groups and the strength of adsorption. To further optimize performance, stimuli-responsive polymers that adjust charge in response to environmental triggers such as pH or redox conditions can be employed [45]. For instance, poly(β-amino esters) become protonated under acidic conditions, enhancing electrostatic adsorption of anionic biomolecules in tumor microenvironments [46]. Biomimetic coatings such as hyaluronic acid and polysialic acid can also be wrapped around NPs to mimic natural interfaces and promote specific electrostatic interactions with receptors [47,48]. Polymer coatings offer a flexible and responsive means to modulate nanoparticle surface charge in dynamic environments, though they may introduce heterogeneity and reduce precision at the molecular scale.

### 3.3. Layer-by-Layer Assembly

Layer-by-layer (LbL) assembly is a versatile and modular technique that involves sequential adsorption of oppositely charged polyelectrolytes onto nanoparticle surfaces [49,50]. Each deposition step reverses the surface charge, enabling the buildup of multiple layers with precise control over thickness, charge density, and surface chemistry. Common polyelectrolytes include positively charged poly(allylamine hydrochloride) (PAH) and negatively charged poly(styrene sulfonate) (PSS), as well as alginate and hyaluronic acid.

LbL coatings can also be designed for dynamic or pH-sensitive behavior, enabling controlled release or reversible adsorption [50,51]. Despite their versatility, LbL systems often suffer from time-consuming fabrication and potential instability under shear stress or dilution in vivo. Additionally, the cumulative thickness may impede the effective diffusion of large biomolecules.

### 3.4. Irradiation-Based Functionalization

Irradiation-based techniques may introduce new functional groups onto nanoparticle surfaces by promoting chemical oxidation or bond breakage. For example, UV-ozone exposure of carbon-based nanoparticles can introduce carboxyl or hydroxyl groups, enhancing negative surface charge [52,53,54]. Direct UV irradiation of SiO_2_ has also been shown to increase surface negativity and potentially influence protein adsorption [55], although a standardized methodology for this approach has not yet been established.

Plasma treatment, including oxygen or ammonia plasma, has been used to generate polar surface functionalities on graphene [56,57]. These treatments are rapid, solvent-free, and tunable, but may require post-functionalization stabilization.

Gamma radiation is primarily used for assisted grafting and the sterilization of nanoparticles [58,59]; however, high-energy irradiation may also be employed to directly modulate the surface charge of nanoparticles, such as diamond nanoparticles [60,61]. Recent findings suggest that gamma irradiation can modify surface properties, reduce zeta potential, and alter surface chemistry in ZnO [62] and SiO_2_ [63] nanoparticles, potentially enhancing electrostatic interactions with oppositely charged biomolecules without the need for chemical additives. However, this approach remains underexplored in the context of drug delivery and needs further investigation.

## 4. Characterization Methods for Nanoparticle–Biomolecule Adsorption

Accurate characterization of biomolecule adsorption on nanoparticles is essential for understanding surface interactions, optimizing functionalization strategies, and predicting the stability of formed nanoparticle–biomolecule complexes. This section outlines the key analytical techniques used to assess nanoparticle–biomolecule adsorption.

### 4.1. Spectroscopic Methods

Spectroscopic techniques are crucial for investigating the chemical composition and conformational changes that occur upon adsorption, particularly with charge-sensitive groups and protein structure.

#### 4.1.1. UV–VIS Spectroscopy

UV–Visible spectroscopy is a rapid and straightforward method for studying the adsorption of biomolecules onto nanoparticles (NPs), particularly plasmonic types such as gold nanoparticles (AuNPs) and silver nanoparticles (AgNPs). These metallic NPs exhibit a characteristic surface plasmon resonance (SPR) peak in the visible region [64,65]. Upon biomolecule adsorption, this peak often undergoes a red shift or broadening due to changes in particle size, surface charge, or aggregation, indicating interaction at the NP interface [66]. The main drawback of this method is its non-specificity [67]; it is challenging to determine whether changes in optical properties are due to biomolecule adsorption or simple nanoparticle aggregation. Therefore, it is best used in combination with complementary analytical techniques.

This method is well suited for monitoring charge-related aggregation in functionalized nanoparticles, particularly after direct chemical or polymer-based modifications that alter interparticle spacing.

#### 4.1.2. FTIR and Raman Spectroscopy

Fourier-Transform Infrared (FTIR) and Raman spectroscopies are both vibrational spectroscopy techniques that may provide valuable insights into molecular interactions of NPs and biomolecules attached. FTIR is based on the principle of absorbance of infrared light, causing the rotation and/or vibration of sample molecules. Detecting specific vibrational modes of chemical groups, such as carboxylates (COO^−^), sulfates (SO_3_^2−^), and hydrogen bonds, as well as amide I and II bands, allows for assessing protein secondary structure (α-helices, β-sheets) [68,69]. When biomolecules adsorb onto nanoparticles, FTIR can reveal whether their conformation is preserved or altered. Shifts in the amide I/II ratio or broadening of bands can indicate structural changes resulting from surface interactions [70,71]. These spectral variations can also be linked to electrostatic interactions or hydrogen bonding between biomolecules and NPs.

Unlike infrared spectroscopy, which measures absorption of infrared light, Raman spectroscopy relies on the scattering of monochromatic light at specific angles. This scattered light provides a “fingerprint” of the molecule’s vibrational modes, offering information on its molecular structure, chemical composition, and bonding. However, conventional Raman spectroscopy may yield a low output signal when analyzing biomolecule adsorption on NPs; thus, Surface-Enhanced Raman Spectroscopy (SERS) is commonly used to identify these adsorptions [72,73]. It is typically used with plasmonic NPs; enhancement is primarily attributed to localized surface plasmon resonance (LSPR), which generates intense electromagnetic fields, “hot spots”, at the nanoparticle surface [74]. This amplification allows SERS to detect low concentrations of analytes with high molecular specificity. Even subtle conformational changes can be detected through shifts in vibrational modes, particularly amide I, III, and side-chain-specific bands [75,76]. However, SERS’s high sensitivity is dependent on nanoparticle aggregation, which can compromise reproducibility [76]. Additionally, intense laser excitation may induce local heating, potentially denaturing sensitive biomolecules.

These techniques are applicable across all major surface functionalization strategies, including silanization, polymer wrapping, and irradiation-based modifications, particularly when new polar or charged groups are introduced.

#### 4.1.3. X-Ray Photoelectron Spectroscopy

X-ray Photoelectron Spectroscopy (XPS) is a surface-sensitive analytical technique used to investigate the elemental composition, chemical states, and electronic environments of atoms at the surface of materials. With a typical probing depth of 1–10 nm, XPS is well suited for characterizing surface modifications of nanoparticles (NPs) and confirming the adsorption of biomolecules [77,78,79]. The adsorption process can be indicated by changes in the N 1s and C 1s spectra, reflecting the formation of a protein layer on the nanoparticle surface [80].

XPS is especially effective in verifying the chemical nature of surface-bound functional groups introduced via silanes, radiation-induced oxidation, or polymer conjugation, providing insight into electrostatic functionality.

### 4.2. Microscopic Methods

Microscopy enables the direct visualization of nanoparticle morphology and biomolecular corona formation, which indirectly reflects electrostatic assembly and spatial organization.

#### 4.2.1. Transmission Electron Microscopy

Transmission Electron Microscopy (TEM) is a high-resolution imaging technique widely used to study the morphology, size, and structural organization of nanoparticles (NPs) [81,82]. TEM can confirm the presence of biomolecular coronas around nanoparticles. Although native biomolecules are typically electron-transparent, contrast can be enhanced using harmful staining agents (e.g., uranyl acetate or phosphotungstic acid), which bind selectively to the organic corona and enable clear differentiation from the inorganic core [83]. The most apparent drawback of this method is the need for drying, which can alter the native conformation of soft biomolecules or lead to artifactual aggregation. Cryo-TEM can mitigate this problem by using rapid vitrification of aqueous samples. This preserves the natural structure and spatial arrangement of nanoparticles and associated biomolecules, allowing for more accurate visualization of soft matter at nanometer resolution [84].

TEM can indirectly reflect changes in surface charge and biomolecule adsorption via corona thickness or aggregation state, especially in systems modified with cationic or anionic polymers.

#### 4.2.2. Atomic Force Microscopy

Atomic Force Microscopy (AFM) is a high-resolution scanning probe technique widely used to characterize surface morphology, roughness, and interaction forces at the nanoscale. In the context of nanoparticle–biomolecule systems, AFM provides both topographical and mechanical insights into adsorption phenomena and is particularly useful for visualizing the effects of surface functionalization [85,86]. Limitations include the need for surface immobilization and potential artifacts from tip–sample interactions.

AFM is particularly effective in visualizing morphological differences arising from surface coatings (e.g., polymer wrapping or UV/ozone-treated surfaces) and detecting charge-induced adhesion forces.

### 4.3. Colloidal and Electrokinetic Methods

These techniques are directly related to surface charge and dispersion behavior, providing essential data on electrostatic attraction, zeta potential shifts, and variations in hydrodynamic size.

#### 4.3.1. Dynamic Light Scattering

Dynamic Light Scattering (DLS) measures the time-dependent fluctuations in the intensity of scattered light caused by the Brownian motion of particles, from which the hydrodynamic sizes are calculated. It is a valuable tool for identifying biomolecule adsorption on nanoparticles, as changes in size reflect the interaction [87,88]. However, the reliability of DLS measurements heavily depends on the monodispersity of the sample. In polydisperse or aggregating systems, it becomes challenging to distinguish between actual adsorption-related size increases and particle aggregation. This issue is particularly acute when the surface charge is modified to enhance electrostatic adsorption, as excessive neutralization can promote interparticle attraction and clustering [89,90]. In low-ionic-strength environments, unscreened electrostatic repulsion, along with the diffuse double layer of counter- and co-ions surrounding the NPs, may interfere with the correlation function analysis. This can result in the appearance of an additional, slower relaxation component—an artifact known as the “slow mode dilemma” [91]. Therefore, the DLS is a powerful tool for detecting size changes due to biomolecule adsorption, but the data should be interpreted cautiously. It is best employed in combination with complementary techniques such as zeta potential analysis and TEM to consider aggregation.

DLS is compatible with nearly all surface modification strategies but is particularly useful for tracking size shifts after polymer or irradiation-induced functionalization, where electrostatic interactions affect hydration layers.

#### 4.3.2. Zeta Potential Analysis

Zeta potential measurements reflect the electric potential at the slipping plane of the electrical double layer surrounding the nanoparticle, and its magnitude indicates the tendency of particles to repel or attract one another. In the context of electrostatic adsorption, zeta potential is commonly used to monitor changes in surface charge following the adsorption of biomolecules [92,93,94]. However, zeta potential does have limitations. It does not directly reveal binding mechanisms or biomolecule identity, and results can be influenced by buffer composition, ionic strength, and the thickness of the adsorbed protein layer [95]. Nevertheless, as a rapid, label-free, and sensitive indicator of surface charge modulation, zeta potential remains a foundational tool in the study of electrostatic adsorption on nanoparticle surfaces.

Zeta potential is a primary indicator of electrostatic modulation following surface modifications, including irradiation-induced oxidation, polymer wrapping, or direct introduction of ionic groups via chemical functionalization.

#### 4.3.3. Differential Centrifugal Sedimentation

Differential Centrifugal Sedimentation (DCS) is a highly sensitive technique for determining nanoparticle size distributions with sub-nanometer resolution. It separates particles based on their sedimentation velocity through a density gradient under high centrifugal forces. When biomolecules adsorb onto nanoparticle surfaces, the resulting increase in hydrodynamic diameter and mass alters the sedimentation profile [96,97,98]. Unlike DLS, DCS offers superior resolution, allowing for the detection of small corona layers and distinguishing between free proteins, NP–protein complexes, and aggregates; however, it is challenging for complexes <5 nm in size [97].

DCS is particularly suited for monitoring subtle changes in sedimentation behavior resulting from thin surface coatings, such as polyelectrolyte layers or biomimetic films applied via layer-by-layer or plasma-assisted methods.

Table 1 below summarizes characterization techniques discussed in Section 4.

No single analytical technique can fully characterize the complex interactions involved in nanoparticle–biomolecule adsorption. Each method offers unique insights, whether structural, chemical, morphological, or electrokinetic, but all come with some limitations, such as low specificity (UV–VIS), sensitivity to aggregation (DLS), sample preparation artifacts (TEM and AFM), or surface-only information (XPS). Therefore, a multimodal approach that combines complementary techniques is essential to gain a comprehensive understanding of adsorption phenomena.

## 5. Conclusions

This review highlights the critical role of electrostatic interactions in nanoparticle–biomolecule adsorption and the diverse surface functionalization strategies developed to enhance these interactions. While approaches such as covalent modification, polymer wrapping, and layer-by-layer assembly offer control over surface charge and binding affinity, each has limitations related to stability, specificity, or synthesis complexity. Notably, while the addition of functional groups is widely studied, the potential of directly altering surface charge, such as through irradiation, without introducing new chemical moieties, remains underexplored and could provide a valuable, reagent-free alternative for tuning surface charge.

Characterizing these complex interactions still requires a combination of techniques, as no single method offers complete insight. Each method reveals specific aspects of adsorption phenomena but is limited in some ways; thus, a multimodal approach remains essential for accurately assessing nanoparticle–biomolecule systems. Future research should prioritize combining complementary methods and exploring non-traditional surface modification techniques to advance nanoparticle-based drug delivery platforms.

## Figures and Tables

**Table 1:** Overview of characterization techniques for nanoparticle–biomolecule adsorption.

Characterization Technique	Information Provided	Advantages	Limitations
UV–VIS Spectroscopy	Optical properties, concentration, hints on agglomeration state	Fast, simple	Low specificity; indirect for binding
FTIR and Raman Spectroscopy	Chemical composition, functional group identification	Detects specific groups and conformational changes; high chemical specificity	Sample heating can be affected by aggregation
X-Ray Photoelectron Spectroscopy (XPS)	Elemental composition and chemical states at the nanoparticle surface	High surface sensitivity; distinguishes functional groups	Surface only
Transmission Electron Microscopy (TEM)	Morphology, size, and corona thickness of nanoparticles	High spatial resolution; can visualize corona formation	Drying may distort structure
Atomic Force Microscopy (AFM)	Surface morphology, roughness, and interaction forces	Nanoscale resolution; detects topographical and mechanical changes	Requires immobilization; possible tip-sample artifacts
Dynamic Light Scattering (DLS)	Hydrodynamic size and distribution of nanoparticles in suspension	Sensitive to size shifts from biomolecule binding.	Affected by aggregation and polydispersity
Zeta Potential Analysis	Surface charge (zeta potential) of nanoparticles in suspensions	Sensitive to surface charge; fast and label-free	Affected by medium properties; indirect for binding
Differential Centrifugal Sedimentation (DCS)	High-resolution particle size distribution and corona layer thickness	High resolution; detects small changes in surface mass	Less effective below ~5 nm

## Data Availability

The data supporting the results in this study are available on request from the corresponding author.
